# TAZ promotes epithelial to mesenchymal transition via the upregulation of connective tissue growth factor expression in neuroblastoma cells

**DOI:** 10.3892/mmr.2014.2818

**Published:** 2014-10-30

**Authors:** QIANG WANG, ZHILIN XU, QUN AN, DAPENG JIANG, LONG WANG, BINGXUE LIANG, ZHAOZHU LI

**Affiliations:** 1 Department of Pediatric Surgery, The First Affiliated Hospital of Harbin Medical University, Harbin, Heilongjiang 150001, P.R. China; 2 Department of Pediatric Surgery, The Second Affiliated Hospital of Harbin Medical University, Harbin, Heilongjiang 150086, P.R. China

**Keywords:** epithelial-to-mesenchymal transition, neuroblastoma, TAZ, connective tissue growth factor, transforming growth factor-β signaling

## Abstract

Neuroblastoma (NB) is a neuroendocrine cancer that occurs most commonly in infants and young children. The Hippo signaling pathway regulates cell proliferation and apoptosis, and its primary downstream effectors are TAZ and yes-associated protein 1 (YAP). The effect of TAZ on the metastatic progression of neuroblastoma and the underlying mechanisms involved remain elusive. In the current study, it was determined by western blot analysis that the migratory and invasive properties of SK-N-BE(2) human neuroblastoma cells are associated with high expression levels of TAZ. Repressed expression of TAZ in SK-N-BE(2) cells was shown to result in a reduction in aggressiveness of the cell line, by Transwell migration and invasion assay. In contrast, overexpression of TAZ in SK-N-SH human neuroblastoma cells was shown by Transwell migration and invasion assays, and western blot analysis, to result in epithelial-mesenchymal transition (EMT) and increased invasiveness. Mechanistically, the overexpression of TAZ was demonstrated to upregulate the expression levels of connective tissue growth factor (CTGF), by western blot analysis and chromatin immunoprecipitation assay, while the knockdown of TAZ downregulated it. Furthermore, TAZ was shown by luciferase assay to induce CTGF expression by modulating the activation of the TGF-β/Smad3 signaling pathway. In conclusion, the present study is, to the best of our knowledge, the first to demonstrate that the overexpression of TAZ induces EMT, increasing the invasive abilities of neuroblastoma cells. This suggests that TAZ may serve as a potential target in the development of novel therapies for the treatment of neuroblastoma.

## Introduction

Neuroblastoma (NB) is a neuroendocrine cancer that occurs most commonly in infants and young children (1). NB tumors are malignant tumors that develop from the nerve cells of an embryo or fetus (2). Certain forms of NB are low risk, while others are high risk and may require multiple treatments.

The Hippo signaling pathway manages organ size by regulating cell proliferation and apoptosis (3,4). Yes-associated protein (YAP) and TAZ are the primary downstream effectors of the Hippo signaling pathway, and these two effectors can be phosphorylated and inhibited by the Hippo pathway kinases LATS1/2 (5,6). There are multiple studies on the correlation between abnormal TAZ activation and human cancers, including breast (7), ovarian (8), colorectal (9), lung (10) and brain cancers (11).

Epithelial-mesenchymal transition (EMT) is a complicated process that leads to the transdifferentiation of epithelial cells into mesenchymal cells (12). EMT is induced by various molecular, cellular, and microenvironmental signals, particularly those caused by the transforming growth factor-β (TGF-β) signaling pathway and its induction of key EMT regulators, including members of the Twist, Zeb, and Snail families of transcription factors (13). Connective tissue growth factor (CTGF) is a secreted matricellular protein that participates in a series of events in cellular proliferation and angiogenesis. Recently, CTGF has been implicated as an important regulator in TGF-β-induced EMT (14).

The effect of TAZ on the metastatic progression of neuroblastoma and the underlying mechanisms involved remain elusive. The aim of the current study was to investigate the effect of TAZ on the expression level of CTGF and consequently its role in the EMT-like metastatic progression of neuroblastoma cells.

## Materials and methods

### Cell culture and transfection

The SK-N-SH and SK-N-BE(2) cell lines were purchased from the Cell Bank of the Chinese Academy of Sciences (Shanghai, China). SK-N-SH was cultured in Dulbecco’s modified Eagle’s medium (DMEM; Invitrogen Life Technologies, Carlsbad, CA, USA) containing 10% fetal bovine serum (FBS; Invitrogen Life Technologies) and SK-N-BE(2) was cultured in advanced DMEM/Nutrient Mixture F-12 (DMEM/F12; Invitrogen Life Technologies) containing 10% FBS. HEK 293 cells were maintained and cultured in DMEM supplemented with 10% FBS. All cell lines were incubated at 37°C in humidified air containing 5% CO_2_.

Cells were transfected with human influenza hemagglutinin (HA)-tagged TAZ or Flag-tagged Smad3 and Smad4 (all OriGene Technologies, Rockville, MD, USA) using Lipofectamine™ 2000 reagent (Invitrogen Life Technologies), according to the manufacturer’s instructions. siRNA oligonucleotides against human TAZ (ON-TARGETplus SMARTpool) and non-targeting siRNAs control oligonucleotides were obtained from GE Dharmacon (Lafayette, CO, USA) and transfected using DharmaFECT1 siRNA transfection reagent (GE Dharmacon).

### Western blot analysis

Whole cell extracts were prepared using Laemmli buffer (Invitrogen Life Technologies). Samples were run on a 10% sodium dodecyl sulfate (SDS)-polyacrylamide gel and transferred to a nitrocellulose membrane (Invitrogen Life Technologies). Membranes were blocked in 10% milk solution [Tris buffered saline with 0.2% Tween 20 (TBST)] for 2 h at room temperature and incubated with the indicated primary antibody overnight at 4°C. The membranes were washed three times for 10 min in TBST at room temperature and incubated for 2 h with the corresponding horseradish peroxidase (HRP)-conjugated secondary antibody (Santa Cruz Biotechnology, Inc., Santa Cruz, CA, USA). Proteins were detected using the enhanced chemiluminiscence system (GE Healthcare Life Sciences, Chalfont, UK) according to the manufacturer’s instructions. The primary antibodies used for western blot analysis were mouse monoclonal anti-TAZ (1:1,000; cat no. ab129153; Abcam, Cambridge, UK), rabbit polyclonal anti-CTGF (1:1,000; cat no. sc-25440), mouse monoclonal anti-GAPDH (1:1,000; cat no. sc-32233; Santa Cruz Biotechnology, Inc.), mouse monoclonal anti-Vimentin (1:1,000; cat no. 550513), mouse monoclonal anti-E-cadherin (1:1,000; cat no. 610182) and mouse monoclonal anti-β-catenin (1:1,000; cat no. 610153; BD Biosciences, Franklin Lakes, NJ, USA).

### mRNA isolation and quantitative reverse transcription polymerase chain reaction (RT-qPCR)

mRNA was isolated using TRIzol^®^ (Invitrogen Life Technologies) and cDNA was prepared using Transcriptor First Strand cDNA Synthesis kit (Roche, Mannheim, Germany) according to the manufacturer’s instructions. The mRNA was reverse-transcribed, and 1/20 volume of the reverse-transcribed product was used for the subsequent qPCR. The primers used for TAZ gene were as follows: forward, 5′-CTTGGATGTAGCCATGACCTT-3′ and reverse, 5′-TCAATCAAAACCAGGCAATG-3′.

### Transwell^®^ migration and invasion assays

Transwell^®^ invasion experiments were performed with 24-well plates with Matrigel™-coated chambers (8 μm pore size) from BD Biosciences. Briefly, cells were allowed to grow to subconfluency (~80%) and serum starved for 24 h. Following detachment with trypsin, cells were washed with PBS, resuspended in serum-free medium and 2×10^4^ cells were added to the upper chamber. Complete medium was added to the bottom wells of the chambers. After 24 h, the cells that had not migrated were removed from the upper face of the filters using cotton swabs, and the cells that had migrated were fixed and stained with crystal violet solution. Cell migration assays were performed similarly, however, without Matrigel™ coating.

### Luciferase assay

HEK 293 cells were cotransfected with the indicated plasmids. A TK-*Renilla* expression plasmid (Promega Corporation, Madison, WI, USA). was used as an internal control. Luciferase activity was measured after 24 h using a Dual Luciferase Assay kit according the manufacturer’s instructions (Promega Corporation). Statistical analysis was performed using GraphPad Prism version 4.0 (La Jolla, CA, USA).

### Chromatin immunoprecipitation (ChIP)

Cells were cross-linked using formaldehyde, the nuclei were isolated and sonicated and the DNA-protein complexes were immunoprecipitated with an anti-HA antibody. The immunoprecipitated DNA was de-cross-linked, digested with proteinase K and purified for PCR amplification. The ChIP-enriched DNA was subjected to PCR using the following CTGF primers: sense, 5′-GGAGTGGTGCGAAGAGGATA-3′, and antisense, 5′-GCCAATGAGCTGAATGGAGT-3′.

### Statistical analysis

The Student’s t-test was used for comparisons between two groups. Comparisons between three or more groups were analyzed with a one-way analysis of variance followed by the Duncan’s test using SPSS version 15.0 (SPSS Inc., Chicago, IL, USA).

## Results

### Expression of TAZ migration and invasion properties of SK-N-BE(2) cells correlate with increasing expression levels of TAZ

**Figure 1:**
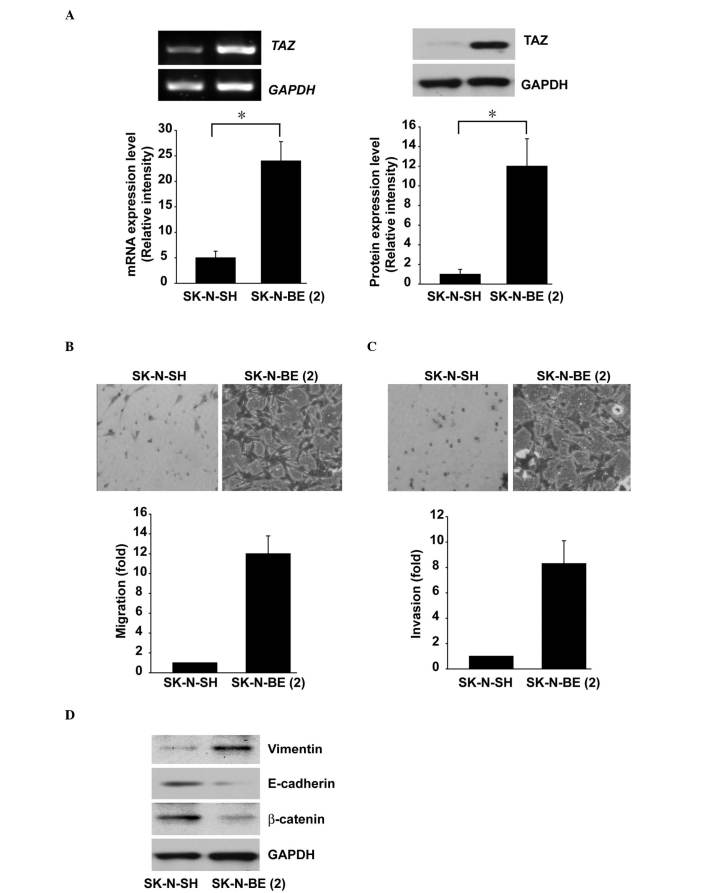
Migratory and invasive properties of SK-N-BE(2) cells expressing high levels of TAZ. (A) The mRNA and protein expression levels of TAZ were higher in SK-N-BE(2) cells compared with those observed in SK-N-SH cells. Top panel: results of reverse transcription quantitative polymerase chain reaction presented as a western blot from a representative study; bottom panel: graph of the relative intensities of TAZ, which were quantified with densitometry and normalized to GAPDH. Values shown are the mean ± standard deviation (SD) from three independent experiments. P<0.05 for SK-N-BE(2) compared with SK-N-SH. (B) SK-N-BE(2) cells had a higher migratory ability than that of SK-N-SH cells, as determined by Transwell^®^ assays. Graph shows the relative fold changes in cell migration compared with SK-N-SH cells. Values shown are the mean ± SD from three independent experiments. Twelve images were randomly captured in each independent experiment. (C) SK-N-BE(2) cells had a greater invasive ability compared with that of SK-N-SH cells, as determined by Transwell^®^ assays. Experiments were performed as described for B. (D) Expression levels of Vimentin, E-cadherin and β-catenin in SK-N-SH and SK-N-BE(2) cells.

The SK-N-BE(2) cell line is a tumorigenic, aggressive and MYCN gene amplified neuroblastoma cell line. The TAZ mRNA and protein expression levels in SK-N-BE(2) cells were significantly higher than those in SK-N-SH cells, which are less aggressive and MYCN gene non-amplified (Fig. 1A). The migration and invasion abilities of SK-N-BE(2) cells, analyzed via Transwell^®^ migration and Matrigel™ invasion assays, were higher than those in the SK-N-SH cells (Fig. 1B and C). These results indicated that TAZ may have a role in neuroblastoma cell migration and invasion. Subsequently, the EMT protein expression levels in the two cell lines were evaluated using western blot analysis. The results revealed that the mesenchymal marker Vimentin was upregulated in the SK-N-BE(2) cells compared with the levels in the SK-N-SH cells; furthermore, the expression levels of the epithelial markers E-cadherin and β-catenin were downregulated in the SK-N-BE(2) cells compared with those of the SK-N-SH cells (Fig. 1D).

### Repression of TAZ expression in SK-N-BE(2) cells results in a decreased aggressiveness of the cell line

**Figure 2:**
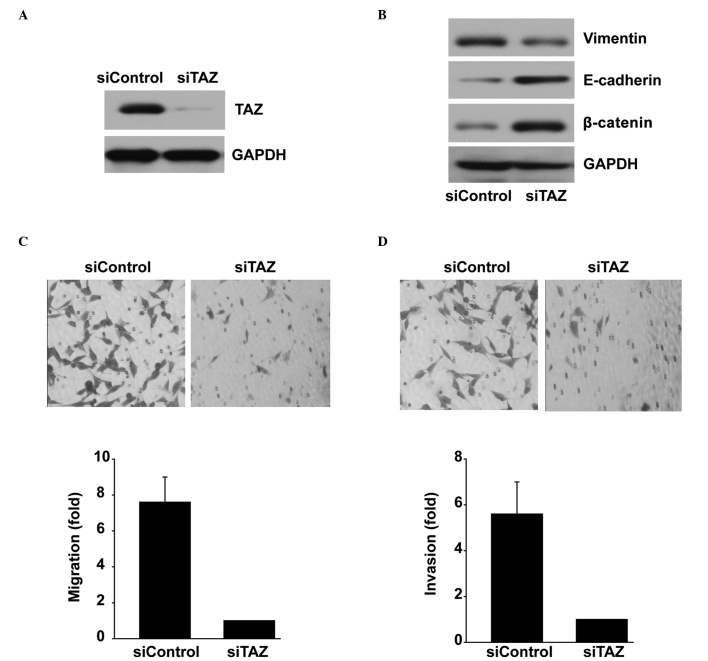
Repressed expression of TAZ in SK-N-BE(2) cells resulted in a reduced aggressiveness of the cell line. (A) The expression of TAZ was decreased through TAZ RNA interference (RNAi). (B) Expression levels of Vimentin, E-cadherin and β-catenin in TAZ-silenced SK-N-BE(2) cells. (C) Compared with the mock control, TAZ RNAi reduced the migratory ability of SK-N-BE(2) cells, as determined by Transwell^®^ assays. (D) Compared with the mock control, TAZ RNAi reduced the invasion ability of SK-N-BE(2) cells, as determined by Transwell^®^ assays.

TAZ was knocked down by siRNA in the aggressive SK-N-BE(2) neuroblastoma cell line, which expresses high levels of TAZ. Knockdown of TAZ in SK-N-BE(2) cells resulted in a marked reduction at the TAZ protein expression level (Fig. 2A). Specific TAZ RNA interference (RNAi) suppressed the protein expression levels of the mesenchymal marker Vimentin. In contrast, the expression levels of the epithelial markers E-cadherin and β-catenin were increased (Fig. 2B). Compared with the mock control, the reduced expression of TAZ via RNAi reduced the migration and invasion abilities of SK-N-BE(2) cells (Fig. 2C and D).

### Overexpression of TAZ in SK-N-SH cells results in EMT and increased invasiveness

**Figure 3:**
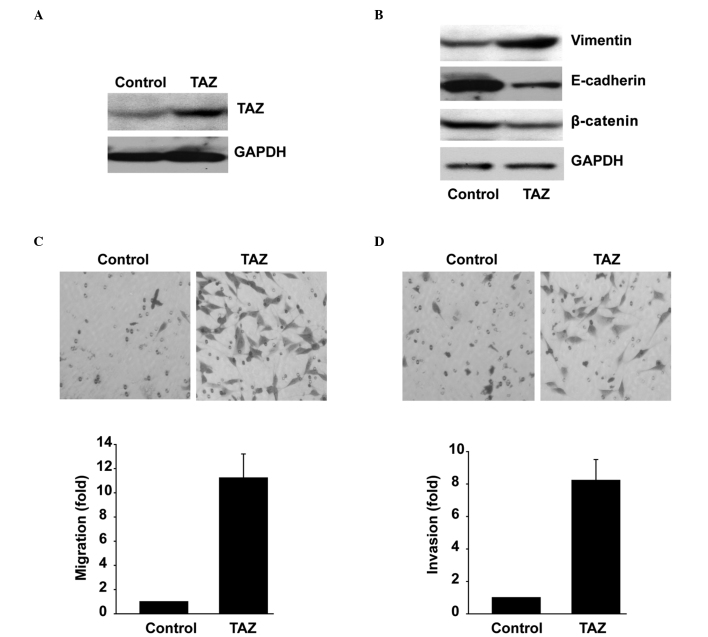
Overexpression of TAZ in SK-N-SH cells results in epithelial-to-mesenchymal transition and increased invasiveness. (A) The expression of TAZ was increased by overexpression of human influenza hemagglutinin-tagged TAZ in SK-N-SH cells. (B) Expression levels of Vimentin, E-cadherin and β-catenin in TAZ-expressing SK-N-SH cells. Compared with the mock control, TAZ overexpression increased the (C) migration and (D) invasion ability of SK-N-SH cells, which was determined by Transwell^®^ assays.

SK-N-SH cells overexpressing TAZ expressed increased levels of the mesenchymal marker Vimentin, and reduced levels of epithelial markers, including E-cadherin and β-catenin, compared with those in the mock control (Fig 3A and B). To determine if the TAZ-induced EMT-like phenotype could be translated into the aggressive ability of the SK-N-SH cells, the migration and invasion of SK-N-SH cells was investigated. SK-N-SH cells overexpressing TAZ showed an increase in motility in the migration and invasion assays, compared with that of the mock control cells (Fig. 3C and D).

### Overexpression of TAZ upregulates CTGF expression but knockdown of TAZ downregulates it

**Figure 4:**
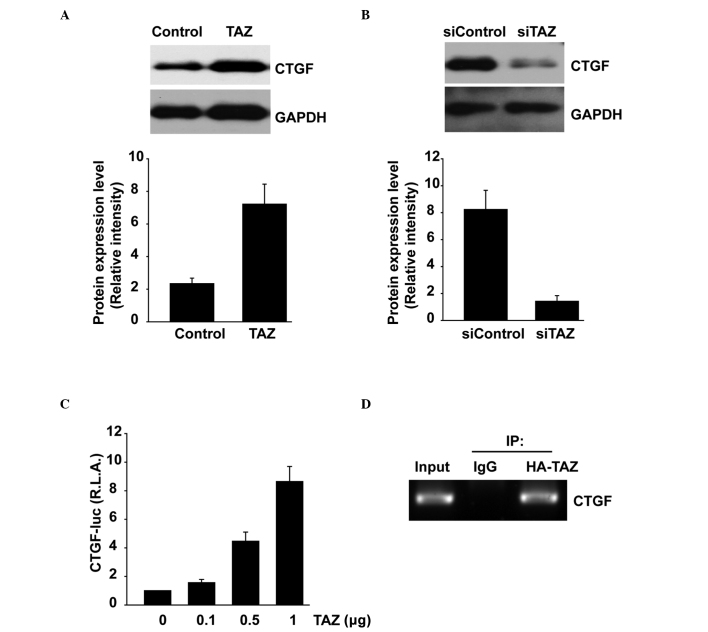
Overexpression of TAZ upregulates connective tissue growth factor (CGTF) expression while knockdown of TAZ downregulates it. (A) Overexpression of TAZ increased the expression levels of CTGF in SK-N-SH cells compared with those in the controls. (B) Knockdown of TAZ repressed the expression of CTGF in SK-N-BE(2) cells compared with that in the control. (C) The indicated plasmids were coexpressed in 293 cells. Cells were harvested for measurement of luciferase activity 24 h after transfection. (D) A chromatin immunoprecipitation assay was performed with anti-human influenza hemagglutinin (HA) antibody using 293 cells expressing HA-TAZ. The presence of CTGF promoter was detected by polymerase chain reaction.

To explore the mechanism by which TAZ induces EMT and increased invasiveness, the regulatory effect of TAZ on the expression of CTGF was investigated. As expected, ectopic TAZ expression clearly induced CTGF at the protein level (Fig. 4A). In contrast, TAZ suppression via RNAi reduced the CTGF expression level compared to that in the controls (Fig. 4B). Subsequently, it was assessed whether TAZ promotes the activity of the CTGF gene promoter. The CTGF luciferase reporter constructs (CTGF-Luc) were transiently transfected into HEK 293 cells with the TAZ expression plasmids. The luciferase reporter activity increased in a concentration-dependent manner in cells with ectopic TAZ expression compared with that in the mock control (Fig. 4C), suggesting that ectopic TAZ expression promotes the activity of CTGF. To determine if TAZ interacted with the endogenous CTGF promoter, ChIP assays were performed. CGTF promoter was detected in PCR-amplified DNA fragments immunoprecipitated with an anti-HA antibody but not in DNA fragments precipitated with an IgG control antibody, indicating that TAZ bound to the CTGF promoter (Fig. 4D).

### TAZ induces CTGF expression via modulating the activation of TGF-β/Smad3 signaling pathway

**Figure 5:**
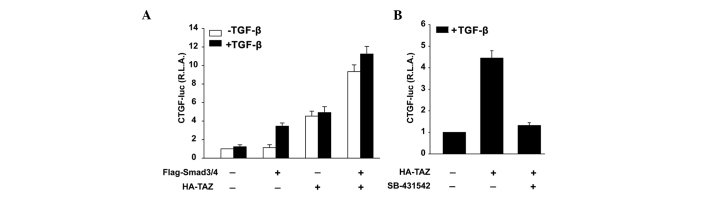
TAZ induces connective tissue growth factor (CTGF) expression via modulating the activation of the transforming growth factor (TGF)-β/Smad3 signaling pathway. (A) Luciferase reporter assay of CTGF promoter constructs in SK-N-SH cells transiently transfected with indicated plasmids and stimulated with 5 ng/ml TGF-β1 (B) Luciferase reporter assay of CTGF promoter constructs in SK-N-SH cells transiently transfected with indicated plasmids and treated with SB-431542 following stimulation with 5 ng/ml TGF-β1.

Smad3 and Smad4 are the two predominant Smads that form complexes in response to TGF-β and are primarily responsible for the EMT phenotype. Cotransfection of Smad3 and Smad4 in SK-N-SH cells overexpressing TAZ led to a strong induction of CTGF promoter activity compared with that of the untransfected cells (Fig. 5A). In contrast, treatment with SB-431542, a specific and selective inhibitor of TGF-β signaling, inhibited the CTGF promoter activity induced by TAZ (Fig. 5B).

## Discussion

TAZ, a 14-3-3 binding protein, can interact with a number of transcription factors (15–17). In the present study, the results demonstrated that TAZ transcriptionally activates CTGF, thereby promoting EMT and invasive progression. Furthermore, this effect is mediated by the TGF-β/Smad3 signaling pathway, as blocking the pathway inhibits TAZ-induced CTGF promoter activity. The expression of TAZ in neuroblastoma cell lines was investigated at the mRNA level by RT-qPCR and at the protein level by western blotting. Compared with SK-N-SH cells, the migration and invasion of SK-N-BE(2) cells was markedly higher which was accompanied by higher expression of TAZ.

EMT is necessary for promoting cancer metastasis (18–20). To demonstrate the role of TAZ in EMT, the expression of TAZ was silenced using siRNA in SK-N-BE(2) cells with highly invasive potential and TAZ high expression. Furthermore, TAZ was overexpressed in SK-N-SH cells with low invasion and TAZ low expression. It was determined that variation in the TAZ expression level correlates with the expression levels of several putative EMT marker. In addition, assessment of the migratory and invasive potential of SK-N-BE(2) cells following transfection with TAZ-siRNA indicated that the rate of cell invasion was markedly reduced compared to those in mock control group, suggesting that TAZ contributes to the migration and invasive potential of neuroblastoma cells.

Although TAZ is thought to contribute to EMT (21), it remains unknown by which molecular mechanism the transcription factor achieves its deleterious effects. It has been established that the TGF-β signaling pathway induces EMT during cancer progression. A recent report revealed that CTGF is required for TGF-β-induced EMT (14). The present study provides a mechanistic explanation in the case of TAZ; i.e., through inducing transcription as exemplified for the CTGF gene.

In conclusion, the present study is, to the best of our knowledge, the first to show that TAZ induces EMT to elicit invasion in neuroblastoma cells. The function of TAZ as an oncogene may be associated with several important molecules involved in the invasion of cancer cells. These results further suggest that TAZ may serve as a potential target for the development of therapies for neuroblastoma.
